# Cloning and functional characterization of the DA_2_ receptor gene in Chinese mitten crab (*Eriocheir sinensis*)

**DOI:** 10.1371/journal.pone.0193999

**Published:** 2018-03-19

**Authors:** Xiaozhen Yang, Genyong Huang, Min-jie Xu, Cong Zhang, Yongxu Cheng, Zhigang Yang

**Affiliations:** 1 National Demonstration Center for Experimental Fisheries Science Education, Shanghai Ocean University, Shanghai, China; 2 Key Laboratory of Freshwater Aquatic Genetic Resources, Ministry of Agriculture, Shanghai Ocean University, Shanghai, China; 3 Shanghai Engineering Research Center of Aquaculture; Shanghai Ocean University, Shanghai, China; Zhejiang University College of Life Sciences, CHINA

## Abstract

Dopamine (DA) plays a modulatory role in numerous physiological processes such as light adaptation and food intake, and exerts these functions through DA receptors (DARs). This study presents, for the first time, isolation and characterization of the dopamine receptor 2(DA_2_ receptor) cDNA from the intestinal tissue of *Eriocheir sinensis*, an economically important freshwater aquaculture species in China. The DA_2_ receptor cDNA sequence, which was obtained by rapid amplification of cDNA ends, is 2369bp long, encode peptide of 589 amino acid, and is highly homologous to related sequences in crustaceans. Analysis of the deduced amino acid sequence and the structure of the DA_2_ indicated that this receptor is a member of the family of G protein-coupled receptors (GPCRs), as it contains seven transmembrane domains and other common signatures of GPCRs. RT-PCR showed that the expression of the DA_2_ receptor gene was distributed in various tissues, and high expression levels were observed in the cranial ganglia and the thoracic ganglia. Further study of the effect of photoperiod on DA_2_ expression showed that constant darkness induced a significant increase in DA_2_ expression in the cranial ganglia. Finally, analysis of DA_2_ receptor expression under different feeding statuses showed that there was significantly greater expression in the hepatopancreas and intestines after feeding than before feeding, but there were no differences in expression between the before feeding and during feeding periods in either tissue. Our results indicate that the DA_2_ receptor structurally belongs to the family of G protein-coupled receptors, and that the cranial ganglia are the main tissues in which the DA_2_ receptor participates in light adaptation during dark hours. In addition, the DA_2_ receptor in *E*. *sinensis* may be involved in the physiological regulation of the hepatopancreas and digestive tract after the ingestion of food. This study provides a foundation for further exploration of the light adaptation and digestive functions of the DA_2_ receptor in decapods.

## Introduction

Dopamine (DA) is a biogenic amine neurotransmitter found in both vertebrates and invertebrates that affects a wide variety of physiological and behavioral functions, including reproduction[1,2], hormone synthesis and release[3], locomotion[4], respiration[5], feeding behavior[6], and the circadian rhythm[7]. The dopamine receptors can be divided into five subtypes (DA_1_-DA_5_), which all belong to the family of G protein-coupled receptors (GPCRs). According to their conserved structures, signaling mechanisms and pharmacological profiles, these receptors are further classified into two types the D1-like and D2-like receptors[1]. The D1-like receptors include the DA_1_ and DA_5_ subtypes, which activate adenylyl cyclase, resulting in increased levels of intracellular cyclic adenosine monophosphate (cAMP); regulate cell metabolism, including ion channel function, and desensitize GPCRs, leading to the release of neurotransmitters. D2-like receptors consist of the DA_2_, DA_3_, and DA_4_ subtypes, which inhibit adenylyl cyclase through the coupled signal transduction pathway and thus decrease cAMP; D2-like receptors can be blocked by the pertussis toxin[8]. In mammals, these receptors occur in the brain, peripheral nervous system, cornea of the eye, heart, kidney and lymphocytes[2]. However, only a few studies on the dopamine receptor in crustaceans have been reported. Using RACE technology and a degenerate PCR strategy with conventional library screening, the gene and protein sequences of DA_1α_, DA_1β_ and DA_2α_ in *Panulirus interruptus* have been obtained[8,9]. In the sequencing of transcriptomes from the nervous systems of *Cancer borealis* and *Homarus americanus*, DA_1α_, DA_1β_ and DA_2α_were found in both decapod crustaceans[10]. In addition, a type 1 dopamine receptor from *Penaeus monodon* has been identified[2]. However, information is still lacking on gene and protein sequences of dopamine receptors in economically valuable decapod crustaceans, such as *E*. *sinensis*, a richly nutritious species with high market demand that has become economically important in Chinese freshwater aquaculture[11].

Light influences the growth and development of crustaceans[12,13], such as *Macrobrachium rosenbergii* and *Portunus pelagicus*, and daily changes in dopamine synthesis and release depend on the interactions between the photoreceptors and the dopaminergic neurons, where dopamine release is induced by light[14,15]. High levels of dopamine have been detected during light periods and low levels during dark periods[16–18], for this reason, it is believed that dopamine promotes light adaptation. However, constant light results in a dramatic reduction in dopamine levels in chicken retina[19]. Constant light and constant darkness have significant effects on survival and growth of larvae of *P*. *pelagicus*[12] and *M*. *rosenbergii*[13], but the effects of different photoperiods on dopamine receptors in crablets remain uncertain.

In addition to promoting light adaptation, dopamine can also participate in feeding regulation. Exogenous injection of DA has been found to significantly decrease food intake compared to that of a control group in neonatal layer-type chickens[6], and cannabinoid-induced feeding behavior may be modulated by dopamine receptor 2[20]. However, by promoting either the initiation or cessation of feeding behavior, increased activity of DA neurons can either increase or reduce food intake[21]; inhibition of D1-type dopamine receptor neurons decreases food intake[22]. Dopamine receptors have also been found to be distributed in the intestinal tract and are considered to be involved in regulating gastrointestinal motility[23,24]. In addition, the presence of specific receptors on the membranes of target cells is essential for dopamine to produce any physiological effects. Inhibitors of dopamine receptors can block the effects of dopamine[25]. Due to the diversity of dopamine receptors, light stimulation has different effects on their expression levels. An understanding of the variation of receptors will help to identify cellular targets of DA and to understand which receptors are activated for particular processes.

The present study describes the molecular cloning and characterization of the dopamine receptor 2 full-length cDNA from *E*. *sinensis* and its expression profile in various tissues under different photoperiods and feeding statuses.

## Materials and methods

### Animals and sampling

In this study, the experimental animals (n = 48) were healthy crablets (exhibiting secondary sexual characteristics) with initial masses of 13.43±1.81 g, collected from the Shuxin crab base in Chongming, Shanghai (China). Crabs were housed for one week for acclimatization in clear glass aquaria (length× width× height = 120×60×40cm) with sufficient ambient medium and cyclic water flow. Crabs were fed once a day at 09:00.

Thirty crabs were acclimatized to 26±1°C and assigned randomly to three groups: a control group (L:D = 12h:12h), a group held in constant darkness (L:D = 0h:24h) and a group held in constant light (L:D = 24h:0h). There were 10 crabs per group, and treatments continued for 14 days[26]. Then crabs from the control group were frozen on ice and dissected, and different tissues, including the gill, heart, muscle, hepatopancreas, intestine, cranial ganglia, thoracic ganglia, eyestalks, and hemolymph were harvested. At the same time, eyestalks, cranial ganglia and thoracic ganglia were harvested on ice from the constant darkness group and constant light group.

For a separate group of 18 crabs, the hepatopancreas and intestine were collected on ice after a week of rearing. Tissues were collected at 08:00 (before feeding, n = 6), 10:00 (feeding period, feeding time was 09:00 to 10:00, n = 6), or 16:00 (after feeding, feces were mostly in the hind gut and the crabs began to evacuate 6h after feeding, n = 6), [27]. All the samples were stored at -80°C until RNA isolation.

### Nucleic acid extraction

Total RNA was extracted from *E*. *sinensis* using the RNAiso Plus reagent (RNA Extraction Kit, TaKaRa, Japan) according to the manufacturer’s instructions. Briefly, tissues were ground in a mortar with liquid nitrogen and collected in 1.5 ml centrifuge tubes. The RNAiso Plus reagent was added (1 ml), and samples were left at room temperature for 5 min. Samples were then centrifuged 5 min at 4°C and 12000 rpm, and the supernatant was collected in new 1.5 ml tubes. Chloroform (200 μl) was added, and samples were then oscillated and again left at room temperature for 5 min before a 15 min centrifugation at 4°C and 12000 rpm. The supernatant was collected in new 1.5 ml tubes and 500 μl of isopropyl alcohol was added. Samples were left at room temperature for 10 min and then centrifuged 10 min at 4°C and 12000 rpm. Pellets were washed with 1 ml of 75% alcohol and centrifuged 10 min at 4°C and 7500 rpm. The supernatant was removed, and the remaining pellets were dried and dissolved in 30 μl of DEPC-treated water. The concentration and quality of the total RNA were estimated by micro-volume ultraviolet-visible spectrophotometer (Quawell Q5000; Thmorgan, China) and agarose-gel electrophoresis, respectively.

### Cloning of full-length *E*. *sinensis* DA_2_ cDNA

Transcriptomes sequences were obtained from the Y-organ of *E*. *sinensis*. The amino acid sequence of the EST (length: 637bp) was verified to be highly homologous to the *C*. *borealis* dopamine receptor 2 (AOG14374.1) using BlastX analysis. A pair of gene primers, DA_2_-F and DA_2_-R (Table 1), was designed to amplify the full-length DA_2_ cDNA from *E*. *sinensis* for sequence verification.

**Table 1:** Primers used in cloning and characterizing the DA_2_ gene.

Primers	Sequences (5’-3’)	Usage
DA_2_-F	CTAGCCATAGTTCTGGCGGCG	RT-PCR
DA_2_-R	TCCTTACCGGACCACAGAACG	RT-PCR
DA_2_-3’Outer	TGAACTCCTTCCTCAACCCCG	3’RACE
DA_2_-3’Inner	GTATGCCAGAGCTAGCCGGG	3’RACE
DA_2_-5’Outer	ATCTTCACTTTCTTCTGCTTCACGA	5’RACE
DA_2_-5’Inner	CTCGTCTGGCTTACGTTCTCGATCAC	5’RACE
qRT-DA_2_-F	TGCTATTATCTGGGTGGTGT	q-RT-PCR
qRT-DA_2_-R	ATGATGAAGTCTGCGTTGTG	q-RT-PCR
18S-F	TCCAGTTCGCAGCTTCTTCTT	q-RT-PCR
18S-R	AACATCTAAGGGCATCACAGA	q-RT-PCR

Four gene-specific primers, DA_2_-3’Outer, DA_2_-3’Inner, DA_2_-5’Outer and DA_2_-5’Inner (Table 1), were designed based on the 637bp singlet to clone the 3’- and 5’-ends of the DA_2_ cDNA by rapid amplification of cDNA ends (RACE) using the SMARTer RACE 5’/3’ Kit (Clontech, USA). The 3’- and 5’-end cDNA templates were synthesized according to the manufacturer’s instructions. Specific products were obtained via touchdown PCR and nested PCR. Touchdown PCR was carried out as follows: 94°C for 5 min; 5 cycles of 94°C for 30 s and 72°C for 3 min; 5 cycles of 94°C for 30 s, 70°C for 30 s, and 72°C for 3 min; 30 cycles of 94°C for 30 s, 68°C for 30 s, and 72°C for 3 min; a final extension for 10 min at 72°C; and a cooling hold at 4°C. Nested PCR amplification conditions were as follows: 94°C for 3 min; 34 cycles of 94°C for 30 s, 66°C for 30 s, and 72°C for 2 min; 72°C for 7 min, and a cooling hold at 4°C. Amplification products were run on a 1.5% agarose gel and purified with a TIANgel Midi Purification Kit (TIANGEN, China). The DNA fragments were cloned into a pMD18-T vector (TaKaRa, Japan) and transformed into TOP10 chemically competent *E*. *coli* cells (TIANGEN, China). Bacteria were grown according to the manufacturer’s instructions. The positive clones containing the inserts of the expected size were sequenced using M13±primers by Sangon Biotech (Shanghai).

### Sequence analysis

The generated sequences were verified for similarity by using the BLAST programs (http://blast.ncbi.nlm.nih.gov/). Then, to obtain the full-length DA_2_ cDNA, the partial fragment, and the 3’- and 5’-end sequences were assembled. After the open reading frame (ORF) was obtained using the ORF finder (http://www.ncbi.nlm.nih.gov/gorf/gorf.html), the coding region sequences were translated into amino acid sequences by using the sequence manipulation suite (SMS) tool (http://www.bio-soft.net/sms/index.html). The molecular mass and the theoretical isoelectric point of the DA_2_ protein were predicted using the Compute pI/Mw tool (http://cn.expasy.org/tools/pi_tool.html). The trans-membrane domains of the protein sequence were predicted by the TMHMM server (http://www.cbs.dtu.dk/services/TMHMM). Protein phosphorylation sites were predicted using DISPHOS 1.3 (http://www.dabi.temple.edu/disphos/). N-glycosylation sites were predicted using the NetNGly 1.0 server (http://www.cbs.dtu.dk/services/NetNGlyc/). An amino acid multiple sequence alignment was performed with the ClustalX program, and phylogenetic tree was constructed using the neighbor-joining (NJ) method with1000 bootstraps in the MEGA 5.0 program[28].

### Real-time quantitative reverse transcriptase-polymerase chain reaction (qRT-PCR) analysis

The mRNA expression pattern of the DA_2_ transcript in various tissues (gill, heart, muscle, hepatopancreas, intestine, cranial ganglia, thoracic ganglia, eyestalks, and hemolymph) were measured by qRT-PCR using 18S ribosome RNA (18S) as a reference gene. We confirmed that 18S expression was stable. Total RNA was extracted from each sample, and reverse transcription was performed with equal quantities of total RNA (1μg). Relative quantification was performed using the ABI 7500 Real-Time PCR System (Life Technology, USA). Gene-specific primers, qRT-DA_2_-F and qRT-DA_2_-R (Table 1), were designed based on the cloned DA_2_ cDNA to produce a 126bp amplicon. Real-time qPCR amplification reactions were performed in a final volume of 10 μl, which contained 5 μl of 2×SYBR Premix Ex Taq^TM^ (TaKaRa, Japan), 1 μl of diluted cDNA template, 3.4 μl of PCR-grade water, 0.2 μl of ROX Reference Dye Ⅱ, and 0.2 μl each of the forward and reverse primers. The PCR conditions used were as follows: 95°C for 30 s; 40 cycles of 95°C for 5 s, and 60 0078C for 34 s; and generation of a melting curve at 95°C for 15 s, 60°C for 1min, and 95°C for 15 s. Samples were run in triplicate, and results were normalized to the expression of the reference gene 18S. The DA_2_ cDNA expression levels were calculated by the 2^-ΔΔCt^ comparative threshold cycle (Ct) method (where ^Δ^Ct = ^Δ^Ct sample - ^Δ^Ct reference). Data were analyzed and presented as triplicate means± SE (standard error) and as n-fold differences relative to the control data.

### Statistical analysis

Statistical analysis of relative gene expression was performed using SPSS software (Chicago, USA; Version 17.0). Data are presented as the means± SE. Statistical significance was determined using one-way analysis of variance and post-hoc Duncan multiple range tests. *P*<0.05 indicated statistical significance.

## Results

### Cloning and identification of the DA_2_ cDNA

**Fig 1:**
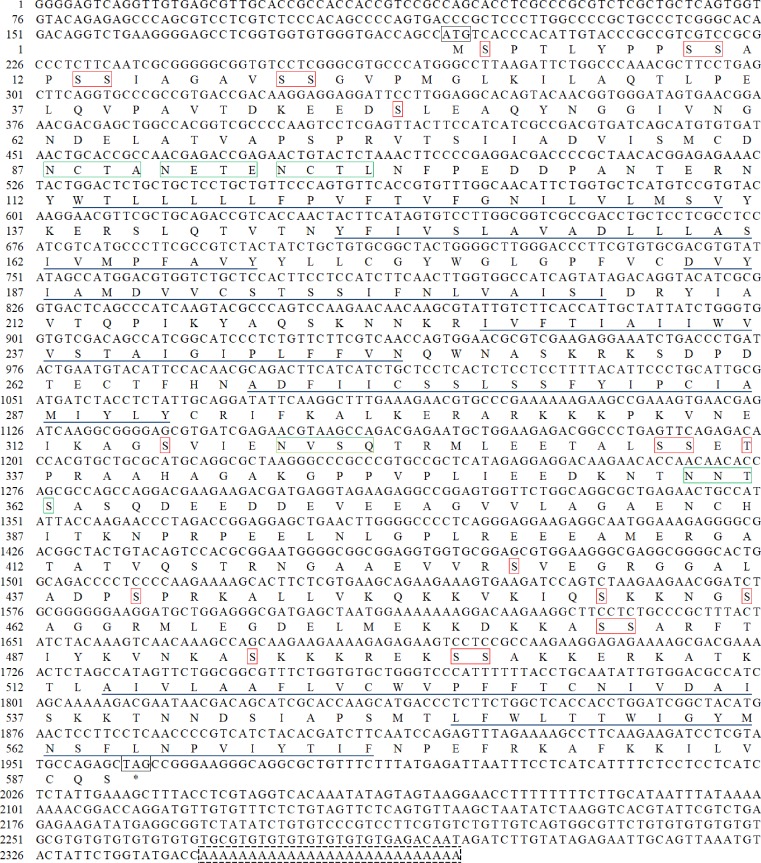
Nucleotide and deduced amino acid sequences of the DA_2_ gene. The nucleotide sequence is enumerated from the 192bp end and the single-letter amino acid code is shown above each corresponding codon. The start codon (ATG) and the stop codon (TAG) are indicated in the black boxes. The putative phosphorylation sites are indicated in the red boxes. The putative N-glycosylation sites are indicated in the green boxes. The seven transmembrane regions are underlined. Dashed boxes indicate the poly (A) tail.

The full-length DA_2_ cDNA was isolated from the intestine of the Chinese mitten crab. The full-length cDNA (2369bp) contained a 1770bp open reading frame (ORF), which encodes a putative DA_2_ protein with 589 amino acids, a 192bp 5’-untranslated region (UTR), and a 407bp 3’-UTR with a 27bp poly (A) tail. Sequence analysis revealed that the DA_2_ protein has a theoretical isoelectric point of 8.37 and a molecular weight of 64.81kDa. The deduced DA_2_ protein has three major domains, four extracellular domains, seven transmembrane domains (TM-Ⅰ through TM-Ⅶ) and four cytoplasmic domains. The transmembrane domains consist of seven hydrophobic regions, which are highly conserved compared with those of other dopamine receptors. In contrast, the amino-terminal region, the second extracellular loop between TM-Ⅳ and TM-Ⅴ, and most of the third cytoplasmic loop between TM-Ⅴ and TM-Ⅵ display a low degree of sequence identity. Despite the low homology between the extracellular N-terminal and the third cytoplamic loops of the *E*. *sinensis* DA_2_ receptor and those of other DA_2_ receptors, consensus motifs for N-linked glycosylation sites (N-x-[S/T]) and consensus sites for phosphorylation by protein kinase C (PKC) (S/T-x-[R/K]) are found in these domains (Fig 1). As in the DA_2_ receptors of other species, there is a conserved DRY motif in the second intracellular loop. Sequence analysis of the DA_2_ cDNA with BLASTn and BLASTp revealed a significant sequence similarity to *C*. *borealis* and *P*. *interruptus* DA_2_ sequences found in the National Center for Biotechnology Information database.

### Homology analysis of the DA_2_ gene

**Fig 2:**
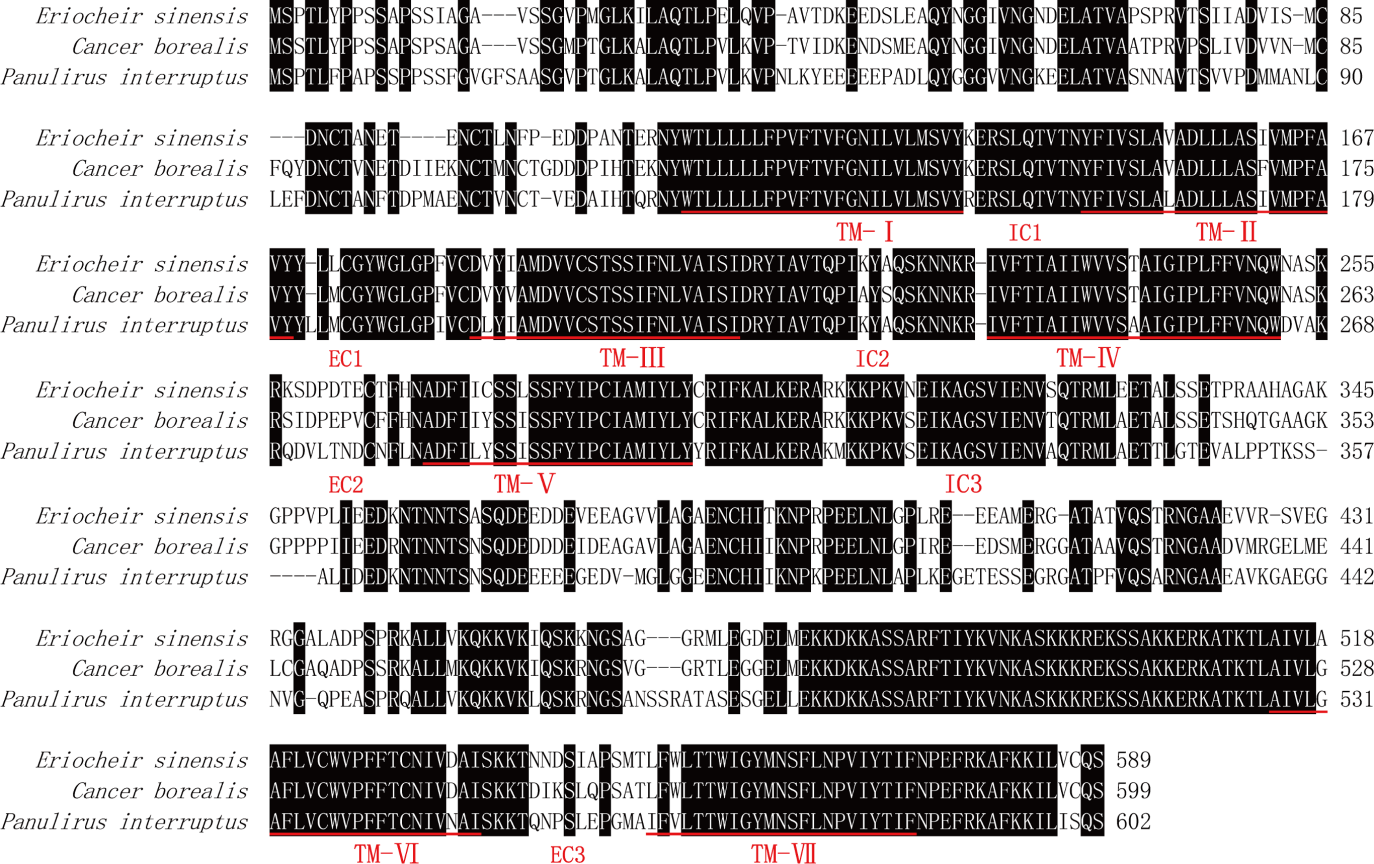
Alignment of amino acid sequences of the DA_2_ receptor from *E*. *sinensis* and other invertebrates. The deduced amino acid sequence of the DA_2_ receptor from *E*. *sinensis* was compared with the DA_2_ receptor sequences from *C*. *borealis* (AOG14374.1) and *P*. *interruptus* (ABI64137.1) with the ClustalX program. Conserved of residues are highlighted in black. The seven transmembrane domains (TM-Ⅰ –TM-Ⅶ), three extracellular loops (EC) and three intracellular loops (IC) are marked.

The degree of homology of the DA_2_ gene with other representative vertebrate and invertebrate DA_2_ amino acid sequences was investigated via multiple sequence alignment in ClustalX (Fig 2). The alignment indicated that the *E*. *sinensis* DA_2_ sequence shares high amino acid identity with the DA_2_ sequence of *C*. *borealis* (84%) and *P*. *interruptus* (72%). The alignment also revealed that the amino acid sequence in the transmembrane domains is highly conserved among these three species.

**Fig 3:**
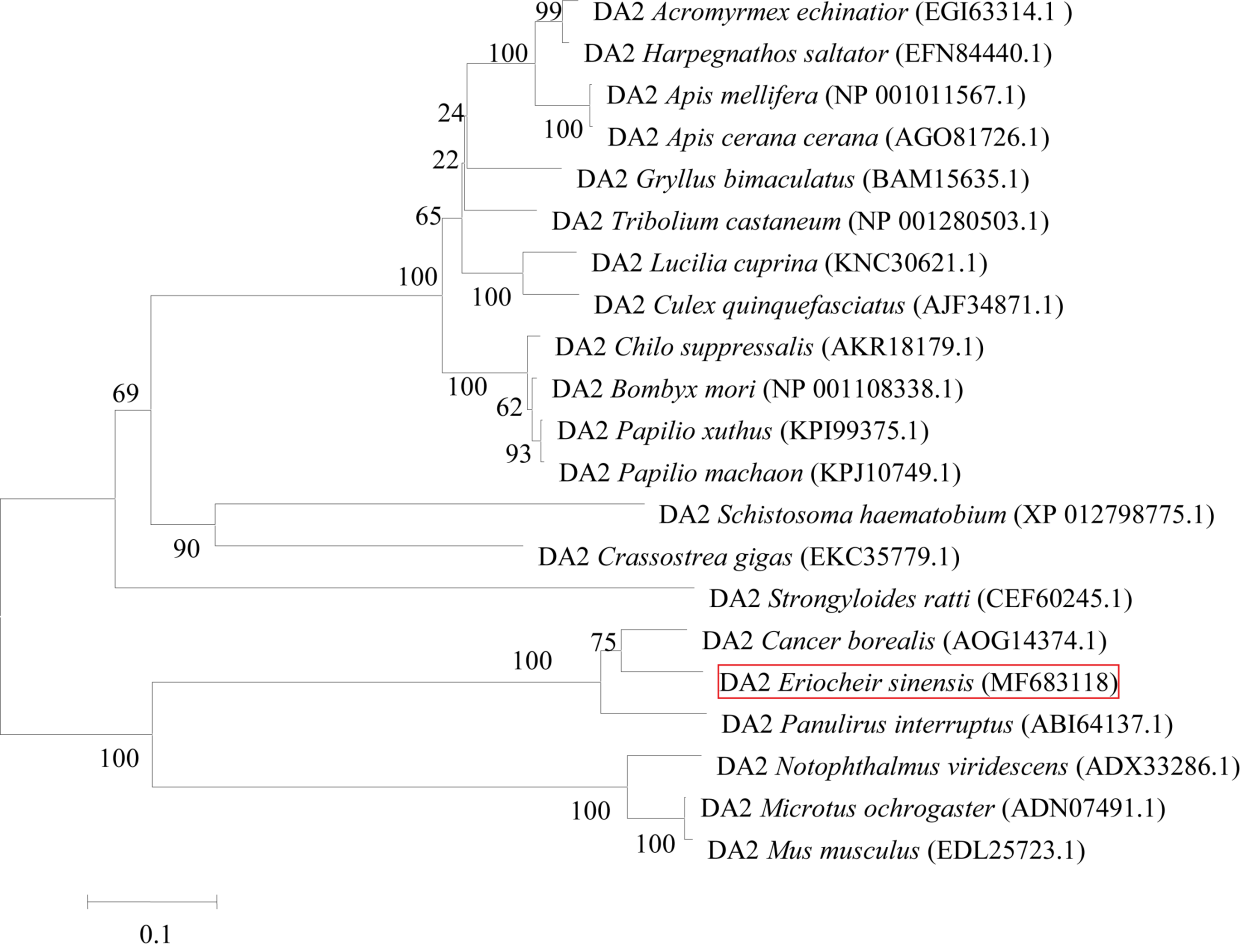
Phylogenetic relationships of the DA_2_ receptor from *E*. *sinensis* and other invertebrates were analyzed with the MEGA 5 program using the neighbor-joining distance analysis. Bootstrap values from 1000 replicates are indicated at the nodes.

A neighbor-joining phylogenetic tree was constructed based on the reported DA_2_ amino acid sequences using MEGA 5.0 software (Fig 3), with confidence in the resulting tree branch topology measured by bootstrapping through 1000 pseudo replicates. The tree provides evidence that the *E*. *sinensis* DA_2_ gene is grouped with DA_2_ genes of other species.

### Tissue distribution of expressed DA_2_ mRNA

**Fig 4:**
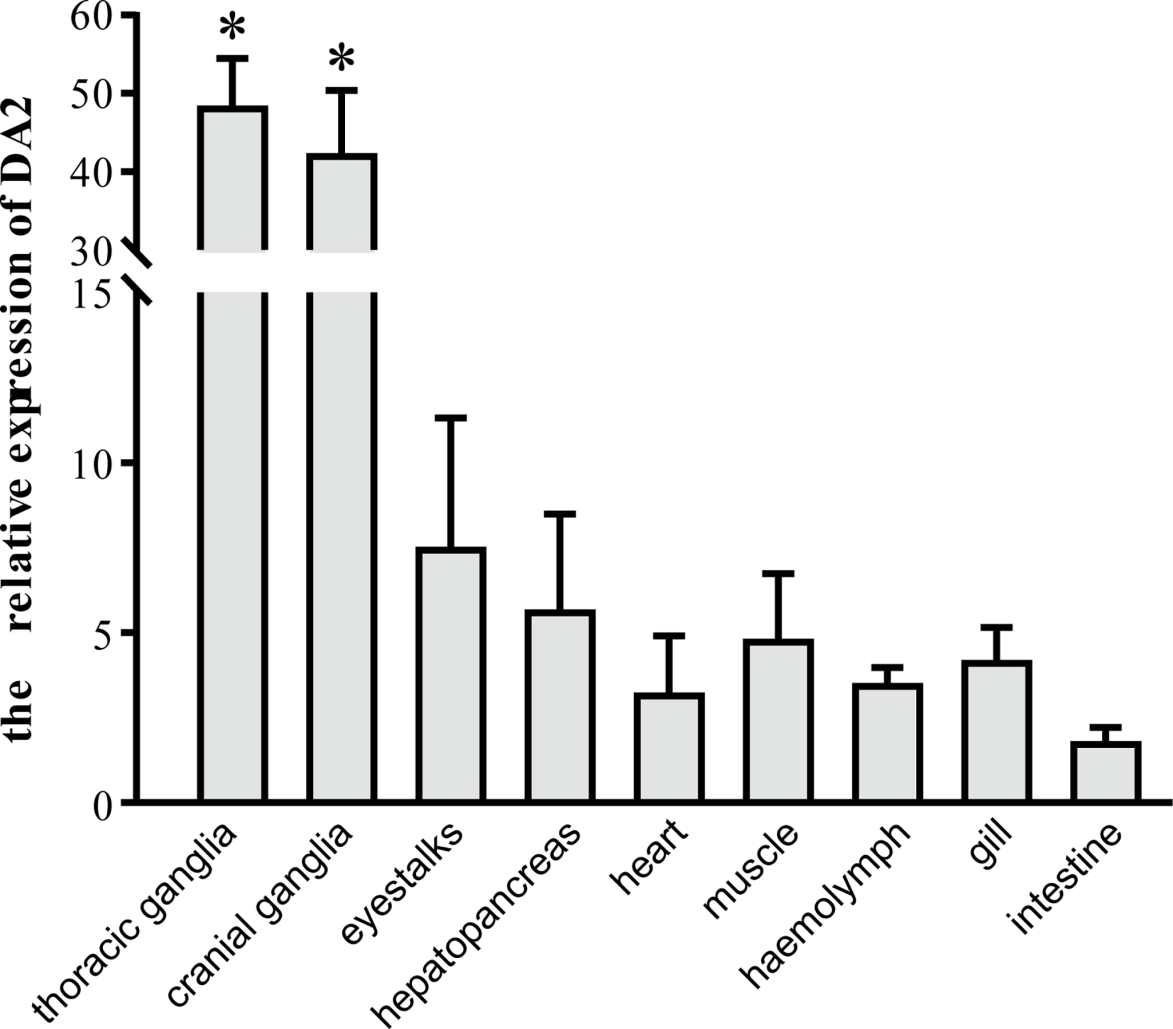
Expression level of the DA_2_ gene normalized to 18S expression in the thoracic ganglia, cranial ganglia, eyestalks, gill, heart, muscle, hepatopancreas, intestine, and haemolymph of the crab. Values are the mean ± SE (n = 4–6). *indicates that expression in the corresponding tissues is significantly different from expression in the control organ (intestine) at *P*<0.05.

To determine mRNA expression patterns of DA_2_ in *E*. *sinensis*, the total RNA extracted from various tissues including gill, heart, muscle, hepatopancreas, intestine, cranial ganglia, thoracic ganglia, eyestalks, and hemolymph was reverse transcribed and subjected to quantitative real-time PCR with qRT-DA_2_-F and qRT-DA_2_-R primers (Table 1). The results (Fig 4) show that DA_2_ mRNA was expressed in all of these tissues, but the expression levels varied. DA_2_ mRNA expression levels were highest in the cranial ganglia and thoracic ganglia (*P*<0.05); only low levels of expression were detected in other tissues (*P*<0.05).

### Effect of photoperiod on DA_2_ mRNA expression in the cranial ganglia and thoracic ganglia

**Fig 5:**
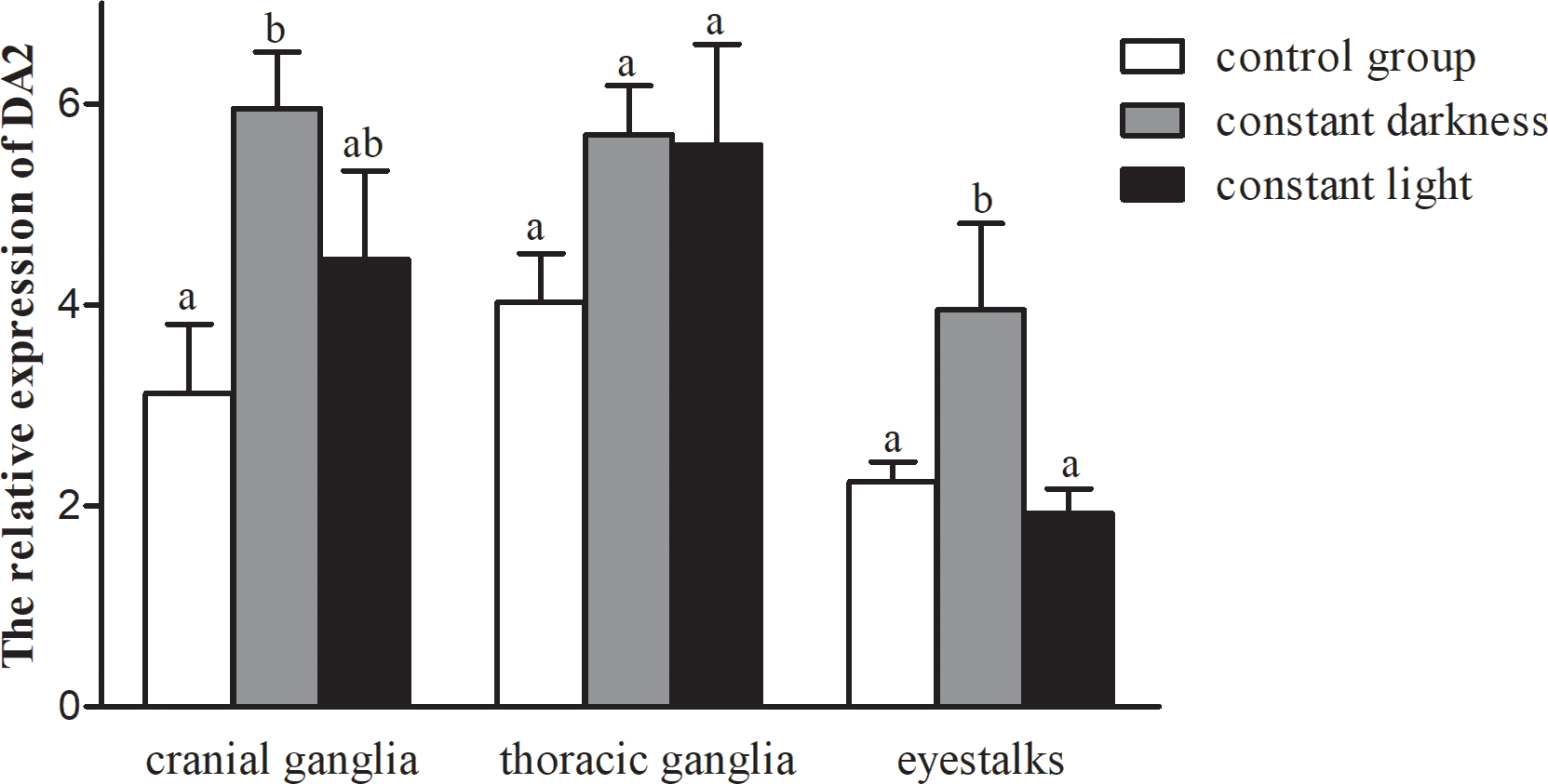
Effect of photoperiod on DA_2_ mRNA expression (normalized to 18S expression) in the cranial ganglia, thoracic ganglia and eyestalks of crabs cultured in different light conditions compared with the control treatment (L:D = 12h:12h). Values are the means± SE (n = 8–10). Bars with different letters indicate statistically significant differences (*P*<0.05).

We determined the effect of photoperiod on DA_2_ mRNA expression in the cranial ganglia and thoracic ganglia after the crabs were cultured in different photoperiods for 14 days (Fig 5). The relative expression of DA_2_ in the cranial ganglia was significantly induced by constant darkness compared with the control treatment (*P*<0.05), while there was no effect of constant light in this tissue. Although the DA_2_ mRNA expression in the thoracic ganglia appeared higher in crabs exposed to constant darkness and constant light than in crabs under control condition, there was no statistically significant difference between treatment (*P*>0.05). As in the cranial ganglia, the expression of DA_2_ in eyestalks was significantly increased by constant darkness (*P*<0.05), but there was no effect of constant light (*P*>0.05).

### DA_2_ mRNA expression levels during different feeding statuses

**Fig 6:**
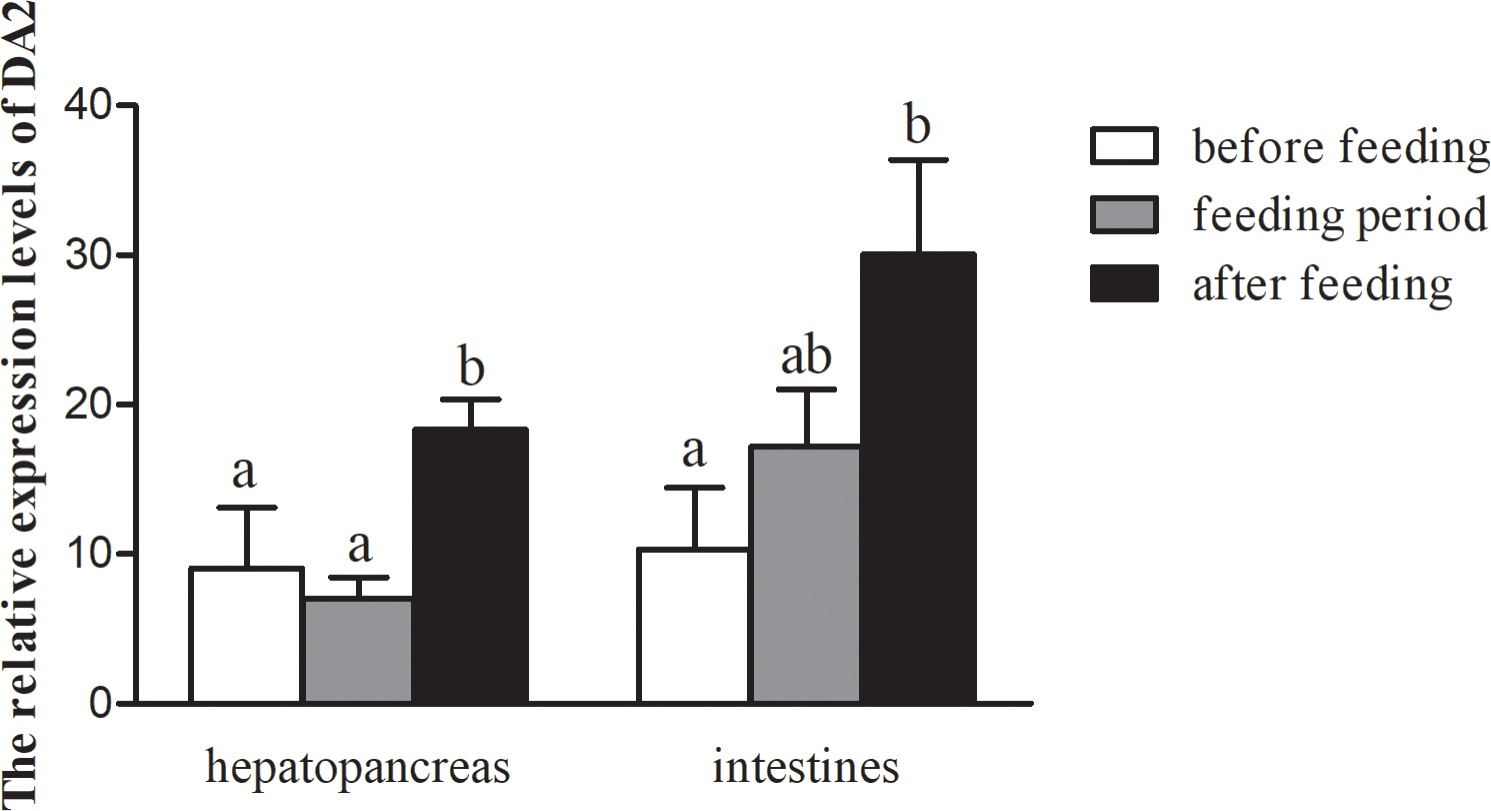
DA_2_ mRNA expression levels in the hepatopancreas and intestines during different feeding statuses. Values are the means± SE (n = 6). Bars with different letters indicate statistical significant differences (*P*<0.05).

To research the relationship between DA_2_ receptors and feeding/digestion, we determined the DA_2_ expression levels in the hepatopancreas and intestines of crabs during three feeding status (Fig 6). In both tissues, the levels of DA_2_ expression were significantly higher after feeding than before feeding (*P*<0.05), but there were no significant differences between the before feeding and during feeding periods (*P*>0.05). In the hepatopancreas, the DA_2_ expression level after feeding was also significantly higher than during the feeding period (*P*<0.05), while in the intestines, the DA_2_ expression level after feeding was not significantly different from expression during with feeding period (*P*>0.05).

## Discussion

In the present study, we have characterized a DA_2_ receptor of the Chinese mitten crab, *E*. *sinensis*. The obtained sequence has considerable similarity with orthologous receptors from other invertebrates[2,8–10,29] and vertebrates[30,31]. The encoded 589 amino acids sequence contains typical characteristics of DA_2_ receptors, such as a large third intracellular loop, a short C-terminal region, a DRY motif in the second intracellular loop and other conserved consensus sequences[32]. Sequence comparison and phylogenetic analysis suggest that DA_2_ is a member of the D-2 like subfamily of dopamine receptors. The DA_2_ in *E*. *sinensis*is most related to the DA_2_ of *P*. *interruptus*, while lower but still remarkable degrees of homology are detected with other arthropods’ DA_2_ receptors; this suggests that the *E*. *sinensis* DA_2_ possesses a highly conserved structure typical of type 2 dopamine receptors.

All residues involved in dopamine receptor activation were present in the *E*. *sinensis* DA_2_ sequence. These include several putative phosphorylation sites and 5 putative N-glycosylation sites, such as those found in mammalian DA_2_ receptors, which are highly phosphorylated and glycosylated neural receptors[31]. In addition, the *E*. *sinensis* DA_2_ contains the conserved DRY (Asp-Arg-Tyr) motif at the interface between TM-Ⅲ and the second intracellular loop; the DRY tripeptide is the key to the conformational changes necessary for receptor activation[33]. The Phe528 in TM-Ⅵ of the DA_2_ is analogous to the conserved phenylalanine in other dopamine receptors that interacts with the aromatic biogenic amine ligand. Two serine residues in TM-Ⅴ (Ser275 and Ser276) are known to be involved in the formation of hydrogen bonds with the catechol hydroxyl group of the dopamine agonist[1]. These interactions aid in the proper positioning of dopamine in the binding pocket of the receptor[34]. On the basis of our analysis, we conclude that the patterns of conservation and divergence observed in the DA_2_ will help to describe the parts of the receptor molecule that are important for proper receptor function[30].

A fundamental aspect of dopamine function in the whole organism is the localization of its receptors in the various areas of the nervous system or in the periphery. When studying DA_2_ gene transcript levels with qRT-PCR, the highest expression was observed in the central nervous tissues, including the cranial ganglia and the thoracic ganglia, followed by the periphery tissues. This result has been supported by several studies showing that the DA_2_ receptor is expressed in the central nervous system and in peripheral tissues. In *Oreochromis niloticus*, higher expression of DA_2_ is found in the anterior part of the brain than in other parts of the brain, and DA_2_ is also expressed in the pituitary gland, liver and gills[31]. high levels of DA_2_ have also been found in the pituitary gland of *Xenopus*[30]. These studies were thus somewhat similar to our results; however, in contrast to our results, the DA_2_ receptor is not expressed in the heart and muscle of the tilapia, *Oreochromis niloticus*. DA_2_ receptors are also expressed by amacrine, bipolar, and ganglia cells[35,36] and possibly by the intrinsically photosensitive retinal ganglion cells[37], functioning as both postsynaptic receptors and autoreceptors that inhibit dopamine release[38]. In addition, the DA_2_ receptor is expressed at high levels in the caudate nucleus, putamen, olfactory bulb, substantia nigra, nucleus accumbens, and ventral tegmental area and is found at low levels in the hypothalamus, kidney, blood vessels, heart, septum, cortex, gastrointestinal tract and sympathetic ganglia in mammals such as rats and humans[23], where it is involved in reward-motivation functions, blood pressure regulation, working memory, and gastrointestinal motility. Thus, overall, DA_2_ receptor primarily present in the nervous system, but also present in peripheral tissues. In the eyestalks and intestines of *E*. *sinensis*, which contain neurons, the expression levels of DA_2_ are similar to levels in the heart, gill and hemolymph; this may be caused by spatial and temporal differences in DA_2_ gene expression. Further research on expression under different environmental conditions is needed to investigate this possibility.

It is well known that DA mediates a various functions via different DA receptors. DA_1_ receptors are involved in coordinating metamorphosis in *Drosophila*[39], and DA_1_ and DA_2_ receptors regulate the phase change of migratory locust in two different directions[40]. In the inner retina, the DA_2_ receptor also plays a role in regulating the development of light responses[41], and in *Daphnia magna*, the DA_2_ receptor is involved in swimming behavior[42]. In the mushroom body of the silkworm, DA_2_ plays a role in the release of the diapauses hormone[29]. In the rat, the DA_3_ receptor may reinforce the effects of cocaine and may be a useful target for treating cocaine abuse[43]. Recent research has shown that DA_4_ activation induces the hippocampal neuronal calcium response[44], and activation of DA_5_ inhibits gastric cancer cell growth[45]. In this study, we researched, for the first time, the effect of photoperiod on DA_2_ mRNA expression.

Light is one of the important environmental factors affecting crustaceans’ survival, directly or indirectly influencing their growth, feeding and reproduction[13,46,47], and light environment is the major factor regulating the synthesis and metabolism of dopamine[16]. As a benthic animal, the Chinese mitten crab prefers a dark environment, and planting waterweed can have a shading and cooling effect that is beneficial to growth and survival[48]. Boosting dopamine can also promote biological growth and survival[49]. In this study, the effect of photoperiod on DA_2_ mRNA expression in the cranial ganglia, thoracic ganglia and eyestalks was analyzed by qRT-PCR. We found that the expression level of the DA_2_ receptor was significantly induced by constant darkness in the cranial ganglia and eyestalks but not affected by constant light in any tissues. Therefore, we speculate that a prolonged dark period is good for the growth of these crabs. Our results are in conformity with Dubocovich’s finding that constant light activates the dopamine-containing retinal neurons, leading to elevated dopamine release and DA_2_ receptor down-regulation[50]. Furthermore, other studies have shown that in the retina, exposure to constant light induces dopamine release and dopamine receptor 2 down-regulation[19,51], and under different light:dark cycles, retinal levels of dopamine are high during light phases and low during dark phases[16]. We believe that the main causes of this difference are species differences and tissue differences. However, there are few studies on the effects of constant darkness on dopamine and dopamine receptors. Therefore, this aspect remains to be thoroughly investigated.

Previous studies have found that feeding status affects glucose metabolism[52], insulin secretion[53] and hypothalamic neuronal activity[54] in vertebrates and that food intake is accompanied by a significant decrease in DA levels in rats compared with levels before food was provided[55]. However, the effect of food intake on DA receptors is not clear. In a previous study, we divided feeding status into 3 categories: before feeding, during the feeding period and after feeding[27]. In this study, we studied the variation of DA_2_ receptor expression in tissues from crabs of different feeding statuses, and the results showed that the expression levels of the DA_2_ receptor were significantly higher after feeding than before feeding or during the feeding period, indicating that the DA_2_ receptor plays an important digestive role in the hepatopancreas and intestines[56]. Although, dopamine hinders the absorption rate of glucose, it seems to play a role in regulating the digestion and transport function of the enterocyte membrane in rats[57]. At the same time, in the hepatopancreas of *Cyrtograpsus angulatus*, DA significantly decreases lipase activity and the digestive capacity was reduced[58]. These findings suggest that there are differential and specific mechanisms by which DA modulates the activity of digestive enzymes in such tissues. Our research on the effects of DA on digestion is of great significance. There have been few studies investigating which types of dopamine receptors play roles in digestion, and therefore these pathway have not yet been elucidated.

## References

[1] Sukthaworn S, Panyim S, Udomkit A. Molecular and functional characterization of a dopamine receptor type1 from *Penaeus monodon*. Aquaclture. 2013; 380-383(1):99–105. doi: 10.1016/j.aquaculture.2012.12.001

[2] Yang BY, Ni JB, Zeng Z, Shi B, You WW, Ke CH. Cloning and characterization of the dopamine like receptor in the oyster *Crassostrea angulata*: Expression during the ovarian cycle. Comparative Biochemistry and Physiology Part B. 2013; 164(3):168–175. doi: 10.1016/j.cbpb.2012.12.006 . 23274282 10.1016/j.cbpb.2012.12.006

[3] Kuo C, Hsu C, Lin C. Hyperglycaemic effects of dopamine in tiger shrimp, *Penaeus monodon*. Aquaclture. 1995; 135(1–3):161–172. doi: 10.1016/0044-8486(95)01011-4

[4] Sakharov DA, Salxnki J. Effects of dopamine antagonist on snail locomotion. Experientia. 1982; 38(9):1090–1091.

[5] Syed NI, Winlow W. Respiratory behavior in the pond snail *Lymnaea stagnalis*. II. Neural elements of the central pattern generator (CPG). Journal of Comparative Physiology A. 1991; 169(5):557–568. doi: 10.1007/BF00193546

[6] Zendehdel M, Moosadoost Y, Masoumi R, Rostami B, Shahir MH, Hassanpour S. Endogenous Nitric oxide and Dopamine regulates feeding behavior in neonatal layer-type chicken. Annals of Animal Science. 2017 doi: 10.1515/aoas-2016-0094

[7] Korshunov KS, Blakemore LJ, Trombley PQ. Dopamine: A Modulator of Circadian Rhythms in the Central Nervous System. Frontiers in Cellular Neuroscience. 2017; 11(91):1–17. doi: 10.3389/fncel.2017.00091 28420965 10.3389/fncel.2017.00091 PMC5376559

[8] Clark MC, Baro DJ. Molecular cloning and characterization of crustacean type-one dopamine receptors: D_1αPan_ and D_1βPan_. Comparative Biochemistry and Physiology Part B. 2006; 143(3):294–301. doi: 10.1016/j.cbpb.2005.11.017 . 16426885 10.1016/j.cbpb.2005.11.017 PMC4019047

[9] Clark MC, Baro DJ. Arthropod D_2_ receptors positively couple with cAMP through the Gi/o protein family. Comparative Biochemistry and Physiology Part B 2007; 146(1):9–19. doi: 10.1016/j.cbpb.2006.08.018 . 17134931 10.1016/j.cbpb.2006.08.018 PMC1868671

[10] Northcutt AJ, Lett KM, Garcia VB, Diester CM, Lane BJ, Marder E, et al Deep sequencing of transcriptomes from the nervous systems of two decapod crustaceans to characterize genes important for neural circuit function and modulation. BMC Genomics. 2016; 17(1):868–889. doi: 10.1186/s12864-016-3215-z . 27809760 10.1186/s12864-016-3215-z PMC5096308

[11] Yang XZ, Zhang C, Huang GY, Xu MJ, Cheng YX, Yang ZG, et al Cellular and biochemical parameters following autotomy and ablation-mediated cheliped loss in the Chinese mitten crab, *Eriocheir sinensis*. Developmental and Comparative Immunology. 2018; 81:33–43. doi: 10.1016/j.dci.2017.11.003 29146453 10.1016/j.dci.2017.11.003

[12] Andrés M, Rotllant G, Zeng CS. Survival, development and growth of larvae of the blue swimmer crab, *Portunus pelagicus*, cultured under different photoperiod conditions. Aquaculture. 2010; 300(1–4):218–222. doi: 10.1016/j.aquaculture.2009.12.026

[13] Lin XT. Influence of phtotperiod on food consumption and development of *Macrobrachium Rosenbergii* larvae. Oceanologia et limnologia sinica. 1997; 28(1):13–20.

[14] Nir I, Haque R, Iuvone PM. Diurnal metabolism of dopamine in dystrophic retinas of homozygous and heterozygous *retinal degeneration slow (rds)* mice. Brain Research. 2000; 884(1–2):13–22. doi: 10.1016/S0006-8993(00)02855-9 . 11082482 10.1016/s0006-8993(00)02855-9

[15] Iuvone PM, Galli CL, Garrison-Gund CK, Neff NH. Light stimulates tyrosine hydroxylase activity and dopamine synthesis in retinal amacrine neurons. Science. 1978; 202(4370):901–902. doi: 10.1126/science.30997 . 30997 10.1126/science.30997

[16] Lorenc-Duda A, Berezińska M, Urbańska A, Gołembiowska K, Zawilska JB. Dopamine in the Turkey Retina—An Impact of Environmental Light, Circadian Clock, and Melatonin. Journal of Molecular Neuroscience. 2009; 38(1):12–18. doi: 10.1007/s12031-008-9153-8 . 18953673 10.1007/s12031-008-9153-8

[17] Shelke RRJ, Lakshmana MK, Ramamohan Y, Raju TR. Levels of dopamine and noradrenaline in the developing retina—effect of light deprivation. International Journal of Developmental Neuroscience. 1997; 15(1):139–143. doi: 10.1016/s0736-5748(96)00080-9 . 9099624 10.1016/s0736-5748(96)00080-9

[18] Doyle SE, McIvor WE, Menaker M. Circadian rhythmicity in dopamine content of mammalian retina: role of the photoreceptors. Journal of Neurochemistry. 2002; 83(1):211–219. doi: 10.1046/j.1471-4159.2002.01149.x . 12358745 10.1046/j.1471-4159.2002.01149.x

[19] Bartmann M, Schaeffel F, Hagel G, Zrenner E. Constant light affects retinal dopamine levels and blocks deprivation myopia but not lens-induced refractive errors in chickens. Visual Neuroscience. 1994; 11(2):199–208. doi: 10.1017/S0952523800001565 . 8003448 10.1017/s0952523800001565

[20] Khodadadi M, Zendehdel M, Baghbanzadeh A, Babapour V. Consequence of dopamine D2 receptor blockade on the hyperphagic effect induced by cannabinoid CB1 and CB2 receptors in layers. British poultry science. 2017; 58(5):585–593. doi: 10.1080/00071668.2017.1357799 . 28728428 10.1080/00071668.2017.1357799

[21] Boekhoudt L, Roelofs TJM, de Jong JW, de Leeuw AE, Luijendijk MCM, Wolterink-Donselaar IG, et al Does activation of midbrain dopamine neurons promote or reduce feeding? International Journal of Obesity. 2017; 41(7):1131–1140. doi: 10.1038/ijo.2017.74 . 28321131 10.1038/ijo.2017.74

[22] Land BB, Narayanan NS, Liu RJ, Gianessi CA, Brayton CE, Grimaldi DM, et al Medial prefrontal D1 dopamine neurons control food intake. Nature Neuroscience. 2014; 17(2):248–253. doi: 10.1038/nn.3625 . 24441680 10.1038/nn.3625 PMC3968853

[23] Ayano G. Dopamine: Receptors, Functions, Synthesis, Pathways, Locations and Mental Disorders: Review of Literatures. Journal of Mental Disorders and Treatment. 2016; 2(2). doi: 10.4172/2471-271x.1000120

[24] Li ZS, Schmauss C, Cuenca A, Ratcliffe E, Gershon MD. Physiological modulation of intestinal motility by enteric dopaminergic neurons and the D2 receptor: analysis of dopamine receptor expression, location, development, and function in wild-type and knock-out mice. Journal of Neuroscience the Official Journal of the Society for Neuroscience. 2006; 26(10):2798–2807. doi: 10.1523/JNEUROSCI.4720-05.2006 . 16525059 10.1523/JNEUROSCI.4720-05.2006 PMC6675162

[25] Khotimchenko YS, Deridovich II. The effect of dopamine and galoperidol on cyclic amp in the gonad of the bivalve mollusc *Mizuhopecten yessoensis* and the sea urchin *Strongylocentrotus intermedius*. Comparative Biochemistry and Physiology Part C 1989; 92(1):23–26. doi: 10.1016/0742-8413(89)90196-5

[26] Tilden AR, Alt J, Brummer K, Groth R, Herwig K, Wilson A, et al Influence of photoperiod on N-acetyltransferase activity and melatonin in the fiddler crab *Uca pugilator*. General and Comparative Endocrinology. 2001; 122(3):233–237. doi: 10.1006/gcen.2001.7641 . 11356035 10.1006/gcen.2001.7641

[27] Li T. Effect of 5-HT on the intestinal motility of Eriocheir sinensis. 2016.

[28] Tamura K, Peterson D, Peterson N, Stecher G, Nei M, Kumar S. MEGA5: molecular evolutionary genetics analysis using maximum likelihood, evolutionary distance, and maximum parsimony methods. Molecular Biology and Evolution. 2011; 28(10):2731–2739. doi: 10.1093/molbev/msr121 . 21546353 10.1093/molbev/msr121 PMC3203626

[29] Mitsumasu K, Ohta H, Tsuchihara K, Asaoka K, Ozoe Y, Niimi T, et al Molecular cloning and characterization of cDNAs encoding dopamine receptor-1 and -2 from brainsuboesophageal ganglion of the silkworm, *Bombyx mori*. Insect Molecular Biology 2008; 17(2):185–195. doi: 10.1111/j.1365-2583.2008.00792.x . 18353107 10.1111/j.1365-2583.2008.00792.x

[30] Martens GJ, Molhuizen HO, Gröneveld D, Roubos EW. Cloning and sequence analysis of brain cDNA encoding a *Xenopus* D2 dopamine receptor. Febs Letters. 1991; 281(1–2):85–89. doi: 10.1016/0014-5793(91)80364-9 . 1826663 10.1016/0014-5793(91)80364-9

[31] Levavi-Sivan B, Aizen J, Avitan A. Cloning, characterization and expression of the D_2_ dopamine receptor from the tilapia pituitary. Molecular and cellular endocrinology. 2005; 236(1–2):17–30. doi: 10.1016/j.mce.2005.03.010 . 15876479 10.1016/j.mce.2005.03.010

[32] Vleugels R, Lenaerts C, Baumann A, Vanden Broeck J, Verlinden H. Pharmacological characterization of a 5-HT_1_-type serotonin receptor in the red flour beetle, *Tribolium castaneum*. PLoS One. 2013; 8(5):e65052 doi: 10.1371/journal.pone.0065052 . 23741451 10.1371/journal.pone.0065052 PMC3669024

[33] Gether U. Uncovering molecular mechanisms involved in activation of G protein-coupled receptors. Endocrine Reviews. 2000; 21(1):90–113. doi: 10.1210/edrv.21.1.0390 . 10696571 10.1210/edrv.21.1.0390

[34] Strader CD, Fong TM, Graziano MP, Tota MR. The family of G-protein-coupled receptors. Faseb Journal Official Publication of the Federation of American Societies for Experimental Biology. 1995; 9(9):745–754. . 7601339

[35] Derouiche A, Asan E. The dopamine D_2_ receptor subfamily in rat retina: ultrastructural immunogold and *in situ* hybridization studies. European Journal of Neuroscience. 1999; 11(4):1391–1402. doi: 10.1046/j.1460-9568.1999.00557.x . 10103134 10.1046/j.1460-9568.1999.00557.x

[36] Nguyen-Legros J, Versaux-Botteri C, Vernier P. Dopamine receptor localization in the mammalian retina. Molecular Neurobiology. 1999; 19(3):181–204. doi: 10.1007/BF02821713 . 10495103 10.1007/BF02821713

[37] Sakamoto K, Liu C, Kasamatsu M, Pozdeyev NV, Iuvone PM, Tosini G. Dopamine regulates melanopsin mRNA expression in intrinsically photosensitive retinal ganglion cells. European Journal of Neuroscience. 2005; 22(12):3129–3136. doi: 10.1111/j.1460-9568.2005.04512.x . 16367779 10.1111/j.1460-9568.2005.04512.x

[38] Tosini G, Iuvone PM. Role of Melatonin and Dopamine in the Regulation of Retinal Circadian Rhythms In: Tosini G, Iuvone PM, Mcmahon DG, Collin SP, editors. Retina and Circadian Rhythms. Springer New York; 2014 p. 49–68.

[39] Regna K, Kurshan PT, Harwood BN, Jenkins AM, Lai CQ, Muskavitch MA, et al A critical role for the Drosophila dopamine D1-like receptor Dop1R2 at the onset of metamorphosis. BMC Developmental Biology. 2016; 16(1):15 doi: 10.1186/s12861-016-0115-z PMID: PMC4868058. 27184815 10.1186/s12861-016-0115-z PMC4868058

[40] Guo XJ, Ma ZY, Kang L. Two dopamine receptors play different roles in phase change of the migratory locust. Frontiers in Behavioral Neuroscience. 2015;9:1–13. doi: 10.3389/fnbeh.2015.00001 PMC4379914. 25873872 10.3389/fnbeh.2015.00080 PMC4379914

[41] Tian N, Xu HP, Wang P. Dopamine D2 receptors preferentially regulate the development of light responses of the inner retina. European Journal of Neuroscience. 2015; 41(1):17–30. doi: 10.1111/ejn.12783 PMID: PMC4331351. 25393815 10.1111/ejn.12783 PMC4331351

[42] Barrozo ER, Fowler DA, Beckman ML. Exposure to D2-like dopamine receptor agonists inhibits swimming in *Daphnia magna*. Pharmacology Biochemistry and Behavior. 2015; 137(101–109. doi: 10.1016/j.pbb.2015.08.010 26296938 10.1016/j.pbb.2015.08.010

[43] Caine SB, Koob GF. Modulation of cocaine self-administration in the rat through D-3 dopamine receptors. Science. 1993; 260(5115):1814–1816. doi: 10.1126/science.8099761 8099761 10.1126/science.8099761

[44] Wang YL, Wang JG, Guo FL, Gao XH, Zhao DD, Zhang L, et al Selective dopamine receptor 4 activation mediates the hippocampal neuronal calcium response via IP3 and ryanodine receptors. Brain Research. 2017; 1670:1–5. doi: 10.1016/j.brainres.2017.05.012 28506554 10.1016/j.brainres.2017.05.012

[45] Leng ZG, Lin SJ, Wu ZR, Guo YH, Cai L, Shang HB, et al Activation of DRD5 (dopamine receptor D5) inhibits tumor growth by autophagic cell death. Autophagy. 2017; 13(8):1404–1419. doi: 10.1080/15548627.2017.1328347 PMID: PMC5584849. 28613975 10.1080/15548627.2017.1328347 PMC5584849

[46] Jiang QC, Zhang WY, Tan HY, Zhang YT, Yang JX, Li F. A study on the feeding rhythm of juvenile Australia red claw crayfish (*Cherax quadricarinatus*) under different photoperiods. Freshwater Fisheries. 2012; 42(5):89–91.

[47] Huang XX, Chen MK, Liu B. The effect of photoperiod on reproduction of the brine shrimp, *Artemia SP*. Acta Hydrobiologica Sinica. 2001; 25(3):297–300.

[48] Zhang Q, Wang ZB, Yang XB, Zhu SH. Effects of the Coverage of Waterweed in Pond on the Growth of *Eriocheir sinensis*. Animal Husbandry and Feed Science. 2014; 35(12):66–67. doi: 10.16003/j.cnki.issn1672-5190.2014.12.029

[49] Van Alstyne KL, Anderson KJ, van Hees DH, Gifford SA. Dopamine release by *Ulvaria obscura* (Chlorophyta): environmental triggers and impacts on photosynthesis, growth, and survival of the releaser. Journal of Phycology. 2013; 49(4):719–727. doi: 10.1111/jpy.12081 . 27007204 10.1111/jpy.12081

[50] Dubocovich ML, Lucas RC, Takahashi JS. Light-dependent regulation of dopamine receptors in mammalian retina. Brain Research. 1985; 335(2):321–325. doi: 10.1016/0006-8993(85)90485-8 . 2988696 10.1016/0006-8993(85)90485-8

[51] De Mello MC, Ventura AL, Paes de Carvalho R, Klein WL, De Mello FG. Regulation of dopamine- and adenosine-dependent adenylate cyclase systems of chicken embryo retina cells in culture. Proceedings of the National Academy of Sciences of the United States of America. 1982; 79(18):5708–5712. doi: 10.1073/pnas.79.18.5708 . 6291061 10.1073/pnas.79.18.5708 PMC346974

[52] Lawrence L, Soderholm LV, Roberts A, Williams J, Hintz H. Feeding status affects glucose metabolism in exercising horses. Journal of Nutrition. 1993; 123(12):2152–2157. . 8263610 10.1093/jn/123.12.2152

[53] Takahashi H, Kurose Y, Sakaida M, Suzuki Y, Kobayashi S, Sugino T, et al Ghrelin differentially modulates glucose-induced insulin secretion according to feeding status in sheep. Journal of Endocrinology. 2007; 194(3):621–625. doi: 10.1677/JOE-07-0206 . 17761901 10.1677/JOE-07-0206

[54] Gautron L, Mingam R, Moranis A, Combe C, Layé S. Influence of feeding status on neuronal activity in the hypothalamus during lipopolysaccharide-induced anorexia in rats. Neuroscience. 2005; 134(3):933–946. doi: 10.1016/j.neuroscience.2005.03.063 . 16039792 10.1016/j.neuroscience.2005.03.063

[55] Meguid MM, Fetissov SO, Blaha V, Yang ZJ. Dopamine and serotonin VMN release is related to feeding status in obese and lean Zucker rats. Neuroreport. 2000; 11(10):2069–2072. doi: 10.1097/00001756-200007140-00002 . 10923645 10.1097/00001756-200007140-00002

[56] Shi OW, Li RH, Mu CK, Song WW, Liu CJ, Wang CL. Optimal conditions for Portunus trituberculatus "Keyong 1" molting. Oceanologia et limnologia sinica. 2015; 46(4):870–878.

[57] Guska NI, Sheptitskiĭ VA, Razlovan TA. The role of dopamine in the mechanism of the regulation of the digestive-transport functions of the enterocyte membrane during stress. Fiziologicheskiĭ Zhurnal Imeni Imsechenova. 1993; 79(6):40–47. 8401653

[58] Michiels MS, Del Valle JC, Mananes AAL. Effect of environmental salinity and dopamine injections on key digestive enzymes in hepatopancreas of the euryhaline crab *Cyrtograpsus angulatus*(Decapoda: Brachyura: Varunidae). Scientia Marina. 2013; 77(1):129–136. doi: 10.3989/scimar.03687.09D

